# Supplementary material for: Integrative Tissue-Specific Functional Annotations in the Human Genome Provide Novel Insights on Many Complex Traits and Improve Signal Prioritization in Genome Wide Association Studies
Source: PLoS Genet. 2016 Apr 8;12(4):e1005947. doi: 10.1371/journal.pgen.1005947 (PMC4825932; doi:10.1371/journal.pgen.1005947)
Supplement: S1 Text — (PDF) [file pgen.1005947.s001.pdf]

Integrative tissue-specific functional annotations in the human genome provide novel insights on many complex traits and improve replication rates in genome wide association studies

Qiongshi Lu[1,#], Ryan Powles[2,#], Qian Wang[2], Beixin J He[3], Hongyu Zhao[1,2,*]

[1] Department of Biostatistics, Yale School of Public Health, New Haven, CT, USA
[2] Program of Computational Biology and Bioinformatics, Yale University, New Haven, CT, USA
[3] Division of Cardiovascular Medicine, Department of Internal Medicine, Yale School of Medicine, New Haven, CT, USA
# These authors contributed equally to this work.

Correspondence: hongyu.zhao@yale.edu

Supplementary Notes

Bimodal pattern in GenoSkyline score

When estimating the proportion of functional genome for each tissue type, we adopted 0.5 as the cutoff for GS score. The GS score histograms for different tissues on chromosome 22 are plotted in **S6 Fig**. The GS score distributions have a clear and consistent bimodal pattern across different tissue types. The similar bimodal pattern can also be observed for other chromosomes. Therefore, the cutoff choice does not substantially affect the estimation of functional proportion.

Robustness of GenoSkyline parameter estimation

The parameters in the GenoSkyline framework were estimated from a set of 12,801,840 nucleotides acquired from GWAS catalog. The reason of using this GWAS-based set is to guarantee the inclusion of a sufficient amount of functional nucleotides. In our previous work, we showed that parameter estimation under this framework is robust [1]. Here, all the 17 parameters were re-estimated after adding 2,000,000 and 6,000,000 bases randomly selected from chromosome 1 to the initial set containing 12,801,840 bases. The parameter estimates remained highly stable (**S11 Table**). These results show that GenoSkyline parameter estimation is insensitive to the choice of the initial set.

Robustness against selection of epigenetic marks

In this section, we compare the blood-specific outcomes of the GenoSkyline framework in the *HBB* region when different epigenetic marks are included in the model. First, we dropped one mark at a time, and then applied the GenoSkyline framework to the

remaining seven annotations. Blood-specific functionality score was then calculated for each nucleotide in the HBB region. In **S7 Fig**, we can see that tissue-specific functionality score remained considerably robust. Most of these seven-mark-annotations were able to effectively capture the known coding and non-coding functional elements in the *HBB* region. However, there was no sign of any seven-mark annotation outperforming the GenoSkyline annotation based on all eight marks. We could also see from **S7 Fig** that H3K4me1 and H3K27ac, two histone marks that are known to associate with enhancer activity, seemed to contribute the most to the GenoSkyline annotation in this region. We then further investigated if functional elements could be identified using only one epigenetic mark. We applied the GenoSkyline framework to one mark at a time and calculated the blood-specific functionality score. Single-mark annotation showed drastic decline in its ability to identify tissue-specific functionality in the *HBB* region (**S8 Fig**). Finally, we compared the results based on two enhancer-associated marks (i.e. H3K4me1 and H3K27ac) with the results based on two promoter-associated marks (i.e. H3K4me3 and H4K9ac). Functional scores based on enhancer-associated marks were much more correlated with known functional elements in the *HBB* region. Interestingly, integrating all eight marks still showed some improvement in identifying functional elements. For example, enhancer-associated marks failed to identify a few cis-regulatory modules in the locus control region while GenoSkyline annotation based on eight marks could identify them with very high resolution (**S8 Fig**).

Since the known functional elements in the *HBB* region were better correlated with the annotations based on enhancer-associated marks than the annotations based on promoter-associated marks, we further investigated if enhancer-associated annotations were also more enriched for GWAS signals using three large-scale GWAS that have strong signal enrichment in blood-specific functional regions (i.e. Crohn's disease, Ulcerative Colitis, and Rheumatoid Arthritis; details of these three studies have been discussed in our manuscript). We analyzed these three GWAS using LD score regression along with its 53 baseline annotations and GenoSkyline annotations of other 6 tissues. The enrichment results for blood-specific annotations based on promoter- or enhancer-associated marks are summarized in **S12 Table**. We could see that blood-specific annotations based on promoter- and enhancer-associated marks were both significantly enriched for GWAS signals for all three diseases. The enrichment in annotations based on promoter-associated marks was consistently less significant than that in annotations based on enhancer-associated marks. However, the fold enrichment was actually substantially stronger in the annotations based on promoter-associated marks. The lower significance level in promoter-mark-based annotation was likely due to its lower coverage of the genome. In fact, 6.1% of the genome was predicted to be functional using enhancer-associated marks while only 1.5% was predicted to be functional based on promoter-associated marks.

In summary, each single epigenetic mark was not sufficient for predicting functional DNA elements. Integrative annotations that combined multiple marks correlated well with the experimentally validated coding and non-coding functional elements in the *HBB* region. Moreover, annotations based on seven marks and annotations based on all eight marks were similar, showing that GenoSkyline is robust against a minor change in the

selection of marks. Finally, annotations based on enhancer-associated marks had better prediction performance in the *HBB* region than functional scores based on promoter-associated marks. Globally, annotations based on promoter-associated marks were still highly significantly enriched for GWAS signals, suggesting that these marks could provide additional insights to complex diseases.

Several remarks on the proportion of functional genome

33.3% of the genome was predicted to be functional based on a GenoCanyon score cutoff 0.5, and the functional proportion was relatively stable across different chromosomes [1]. Since GenoCanyon score has a bimodal distribution, the functional percentage estimate was also robust against this cutoff choice. In this manuscript, we showed that the functional percentages range from 5% to 10% across seven tissue types and their union covers 22.2% of the genome. In this section, we investigate if functional regions in other tissue types could explain the gap between 22.2% and 33.3%.

First, we applied the GenoSkyline framework to eight epigenetic marks for all 111 cells. Functionality score was calculated for each nucleotide on chromosome 22. 33.4% of chromosome 22 was predicted to be functional based on cutoff 0.5. This estimate of functional percentage was highly consistent with the estimate based on GenoCanyon annotation (38.3%) for chromosome 22. The remaining gap may be further explained by the non-tissue-specific annotation data included in the GenoCanyon model (e.g. conservation).

Next, we defined an annotated category "Gap" that included all the regions that were predicted to be functional by GenoCanyon but showed no functionality in any of the seven GenoSkyline annotations. We then analyzed three large-scale GWAS (i.e. height, schizophrenia, and Crohn's disease; details of these three studies have been discussed in the manuscript) using LD score regression along with its 53 baseline annotations, seven GenoSkyline annotations, non-functional genome based on GenoCanyon, and the "Gap" annotation defined above. The enrichment results for GenoSkyline, non-functional genome, and gap annotations are summarized in **S13 Table**.

The "Gap" category was depleted for signals of schizophrenia and Crohn's disease, and enriched for signals of height GWAS. None of these enrichment or depletion was significant. Since GS-brain and GS-blood annotations were highly significantly enriched for signals of schizophrenia and Crohn's disease, respectively, the depletion in the "Gap" category showed that it most likely does not contain important functional elements for brain or blood tissue. Height is a trait that is known to be highly polygenic and hundreds of associated variants have been identified. It is not surprising to see the "Gap" category to be moderately enriched for height signals. Interestingly, the non-functional category based on GenoCanyon annotation was highly significantly depleted of GWAS signals in all three studies. The fold enrichment in non-functional genome was also consistently lower than that in the "Gap" category.

In summary, we did two different analyses to see if the gap between GenoCanyon non-tissue-specific annotations and GenoSkyline annotations of seven tissue types were due to functionality in other tissue types. First, we were able to fill in such a gap by integrating eight epigenetic marks for all 111 Roadmap cells. Second, the gap region was enriched for height GWAS signals and showed consistently stronger enrichment than the non-functional genome. These results showed that it is reasonable to interpret the gap between GenoCanyon and GenoSkyline as potential functional regions in other tissues.

Several remarks on GSP score

For GSP score calculation, we used the mean GS score of the surrounding 10,000 bases as the prior probability $P(Z_T = 1)$ for each SNP. This is because the nucleotide-level GS score may be insufficient for GWAS signal prioritization. In fact, each SNP in GWAS carries information of its nearby variants that are not genotyped or imputed. A better-informed metric needs to measure the functional potential for the surrounding region of each SNP. We chose 10,000 bases as the window size, but no substantial difference in empirical performance was observed when changing the window size to 5,000 or 20,000. In our implemented GenoWAP software for SNP prioritization (available at http://genocanyon.med.yale.edu/GenoSkyline), the users are allowed to use their own annotation data. Therefore, the window size can be changed when necessary. Since the mean GS score of surrounding regions was used as the prior, our SNP prioritization approach is in fact a region-based tool. We emphasize again that it identifies regions of likely functionality and substantially improves the resolution of GWAS, but does not provide conclusive proof of functionality for any individual SNP or locus.

In order to calculate the GSP score, we assumed that SNPs that are functional in a tissue not relevant to the phenotype would have similar p-value behavior to all other SNPs that are not relevant to the phenotype, which in turn behave similarly to SNPs that are not functional at all (see equation 7 in **Methods**). This assumption may not hold exactly due to some intrinsic differences between SNPs located in non-functional regions (Z=0) and those in non-specific functional (Z=1) and tissue-specific functional regions (Z_T=1). As far as we are aware, the main possible contributing factor may be different linkage disequilibrium (LD) patterns in those regions with Z = 0 and those with Z = 1 or Z_T=1. For example, if there is stronger LD in Z_T = 1 regions, then the markers with Z_D = 0 and Z_T = 1 may have a different p-value distribution from those with Z = 0.

In order to check if this is a serious issue, we compared the LD patterns in regions with Z = 0, Z =1, and Z_T = 1 for multiple tissue types on chromosome 22. We downloaded the pre-calculated LD scores [2] for the 1000 Genomes European population from the LD score GitHub page (https://github.com/bulik/ldsc/wiki/LD-Score-Estimation-Tutorial). Based on cutoff 0.1 for GenoCanyon and GenoSkyline scores, we divided all the SNPs on chromosome 22 into tissue-specific functional, non-specific functional and non-functional subcategories. The kernel density estimates of the two subgroups are plotted in **S9 Fig**. It can be seen that there is no substantial difference of LD score distributions between different categories. Therefore, it is reasonable to assume the LD patterns in

regions with $Z = 0$, $Z = 1$, and $Z_T = 1$ to be similar. Moreover, as $Z_D = 1$ is a relatively small proportion of $Z = 1$, this assumption is likely to be a good approximation.

Supplementary References

1. Lu, Q., et al., *A Statistical Framework to Predict Functional Non-Coding Regions in the Human Genome Through Integrated Analysis of Annotation Data.* Sci. Rep., 2015. **5**.
2. Bulik-Sullivan, B.K., et al., *LD Score regression distinguishes confounding from polygenicity in genome-wide association studies.* Nature genetics, 2015. **47**(3): p. 291-295.
