# Supplementary figures and images for: Integrative Tissue-Specific Functional Annotations in the Human Genome Provide Novel Insights on Many Complex Traits and Improve Signal Prioritization in Genome Wide Association Studies
Source: PLoS Genet. 2016 Apr 8;12(4):e1005947. doi: 10.1371/journal.pgen.1005947 (PMC4825932; doi:10.1371/journal.pgen.1005947)

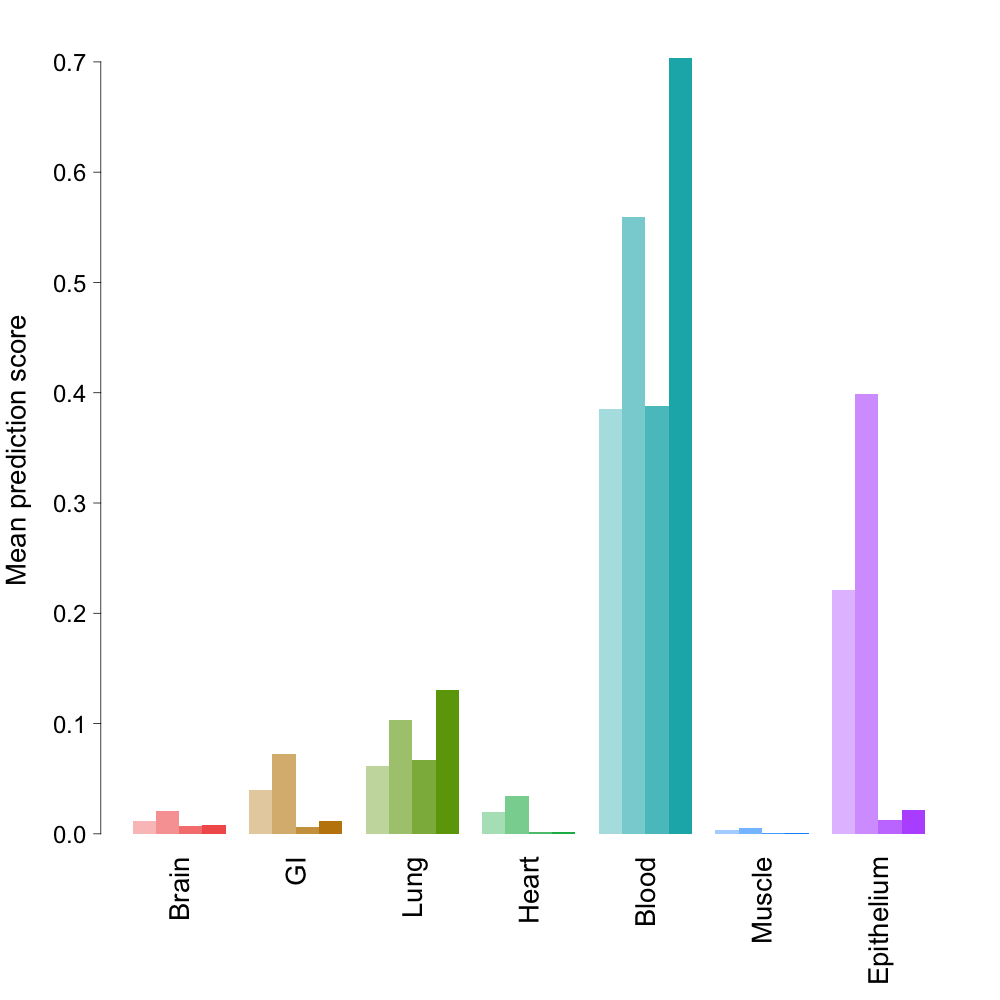

Supplement: S1 Fig — For each tissue, the four bars from left to right indicate all 23 CRMs, adult CRMs, all genes, and adult globins, respectively. (TIFF) [file pgen.1005947.s002.tiff]

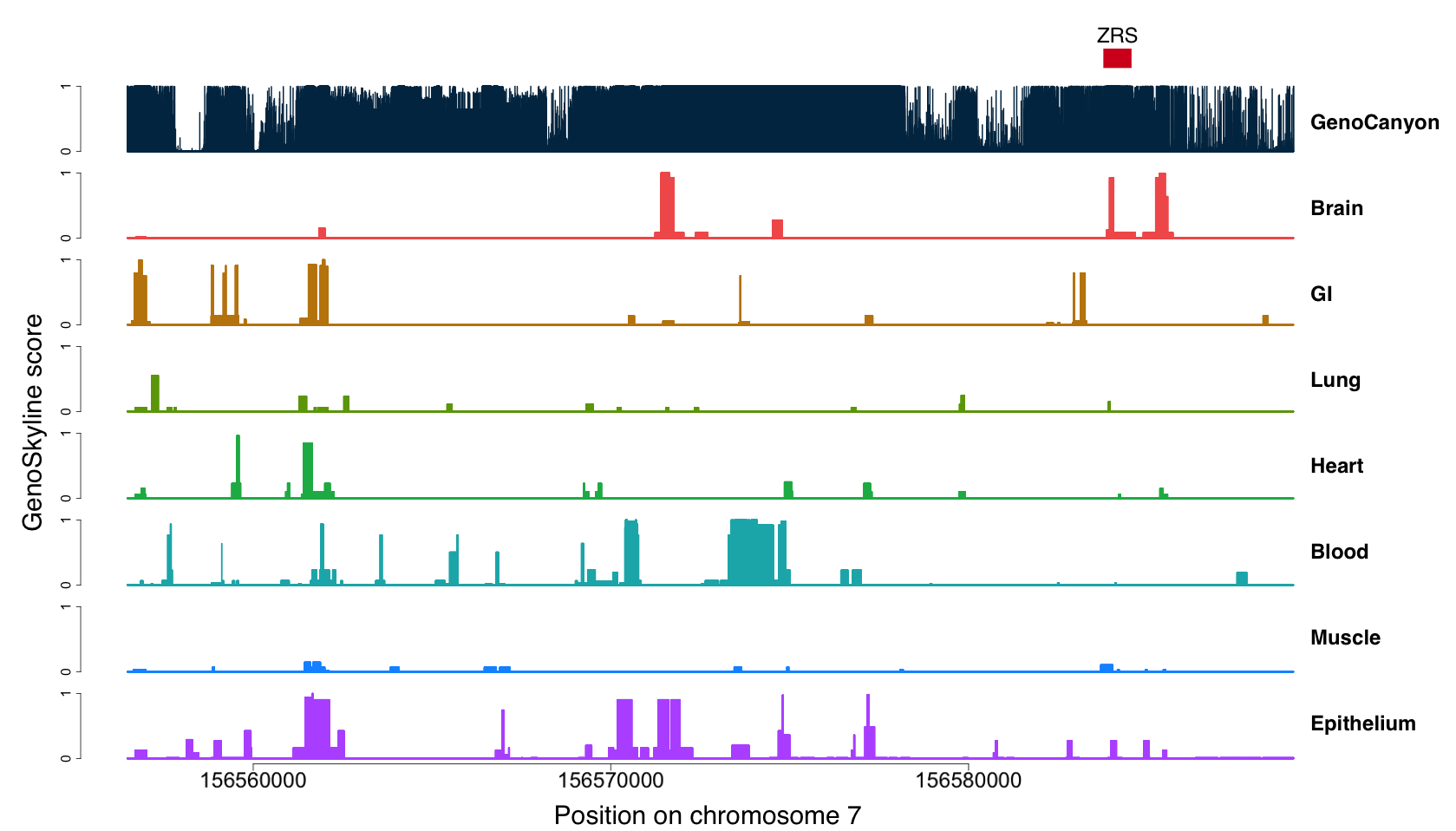

Supplement: S2 Fig — The red box marks the location of ZRS. (TIFF) [file pgen.1005947.s003.tiff]

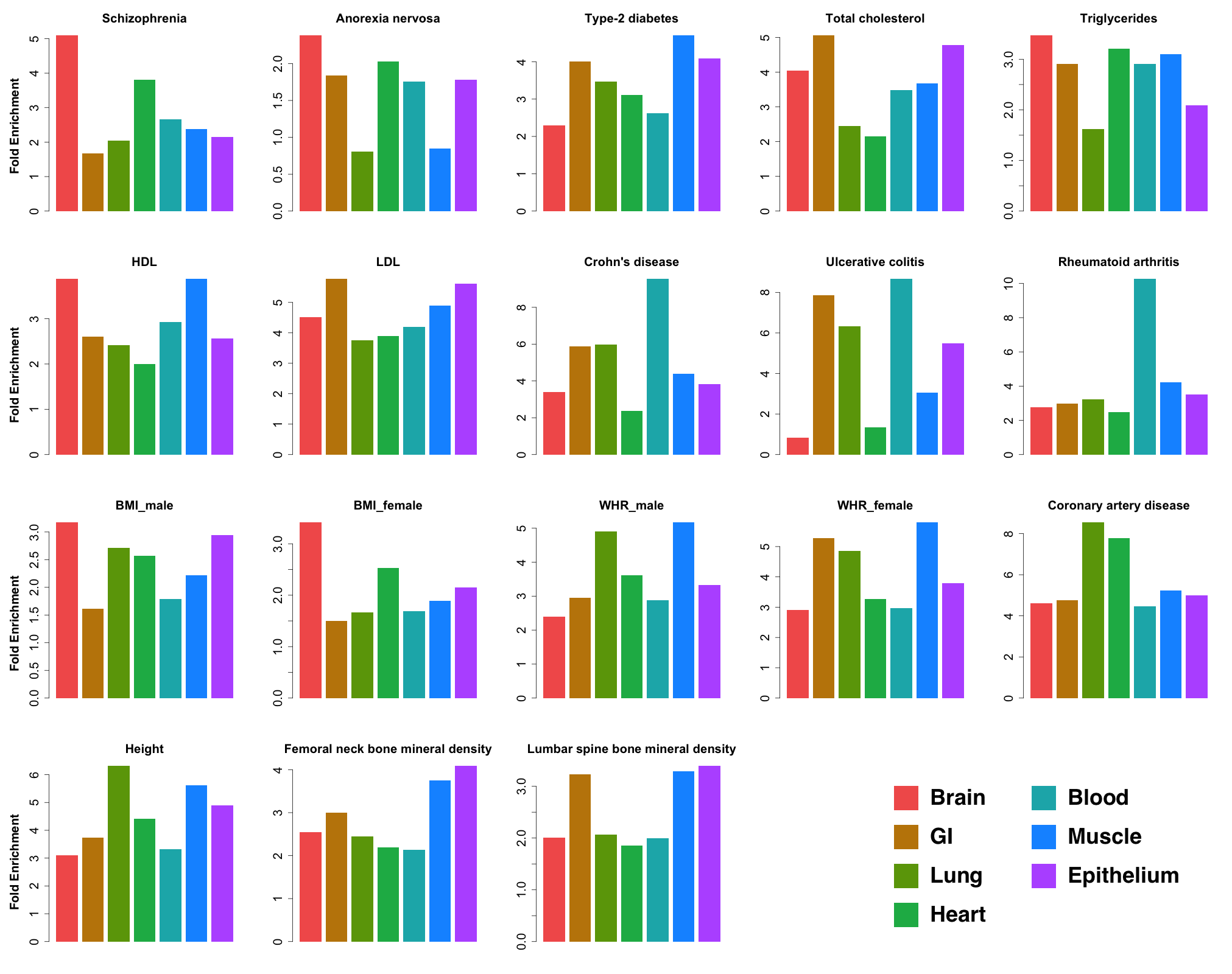

Supplement: S3 Fig — (TIFF) [file pgen.1005947.s004.tiff]

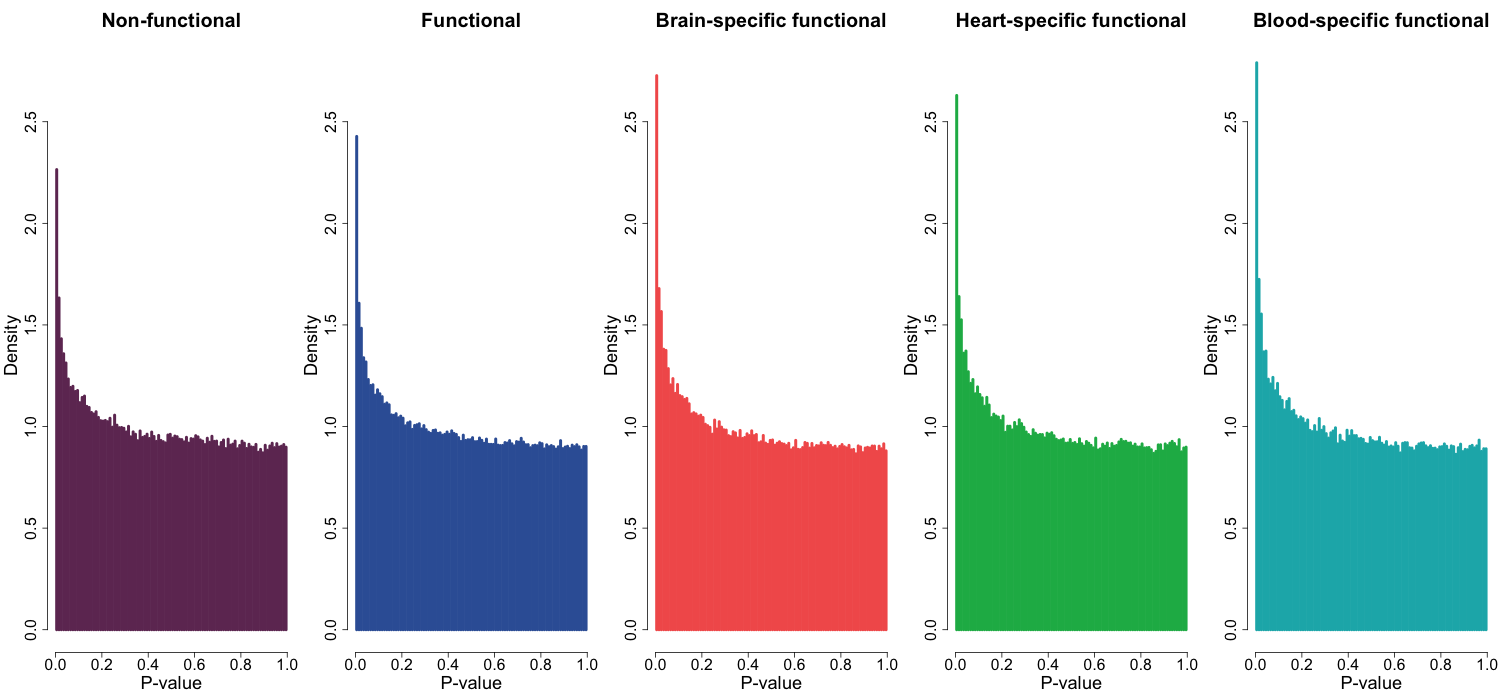

Supplement: S4 Fig — (TIFF) [file pgen.1005947.s005.tiff]

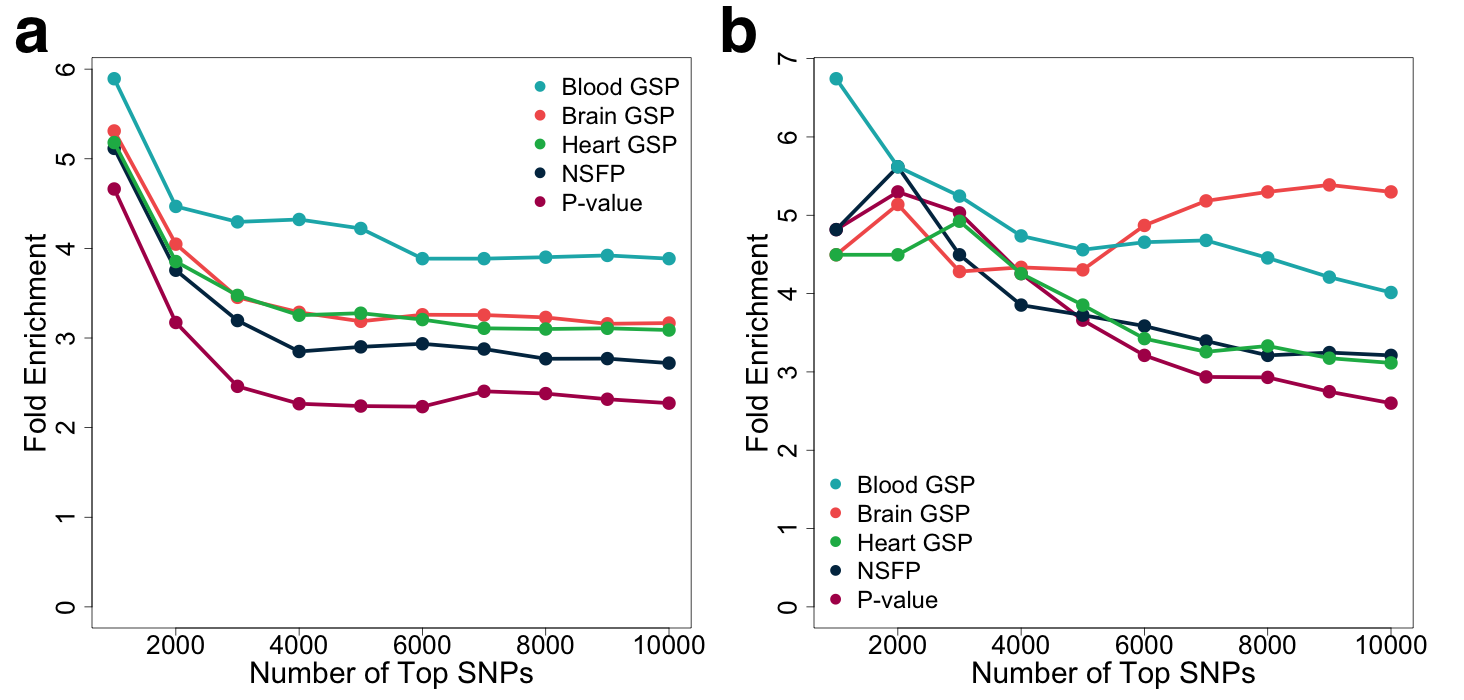

Supplement: S5 Fig — (a) GTEx whole-blood eQTLs. (b) Human brain quantitative trait loci. (TIFF) [file pgen.1005947.s006.tiff]

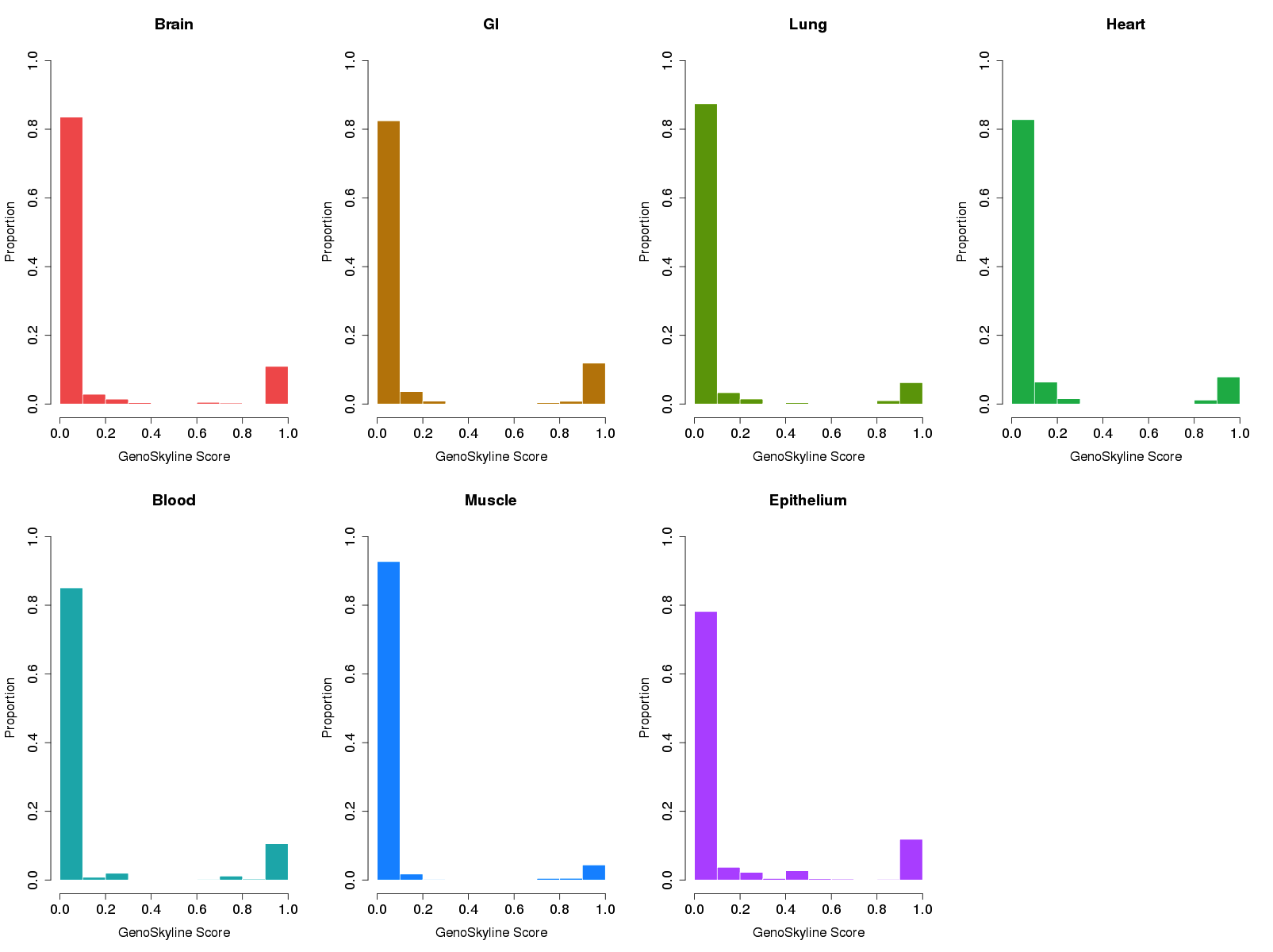

Supplement: S6 Fig — (TIFF) [file pgen.1005947.s007.tiff]

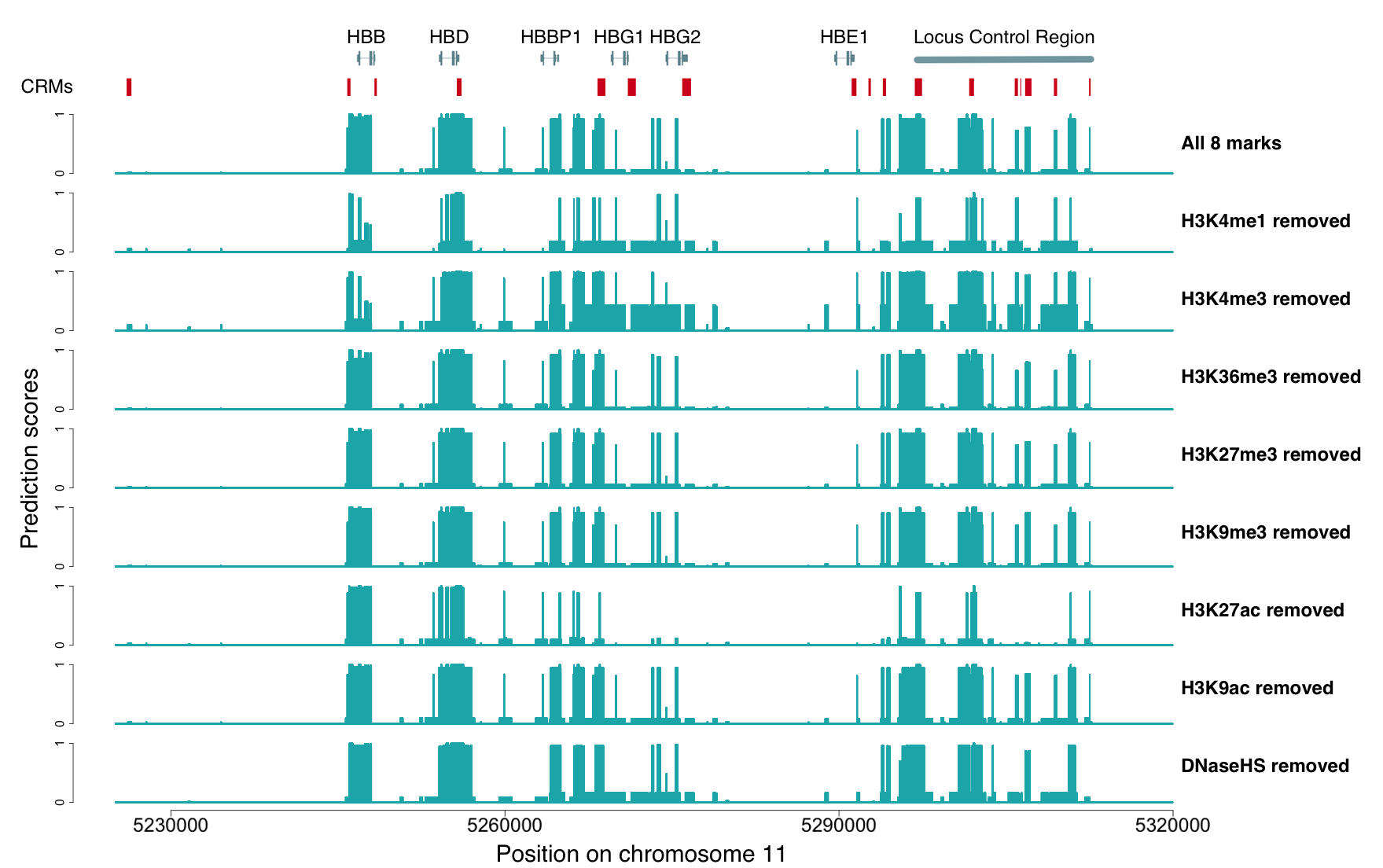

Supplement: S7 Fig — (TIFF) [file pgen.1005947.s008.tiff]

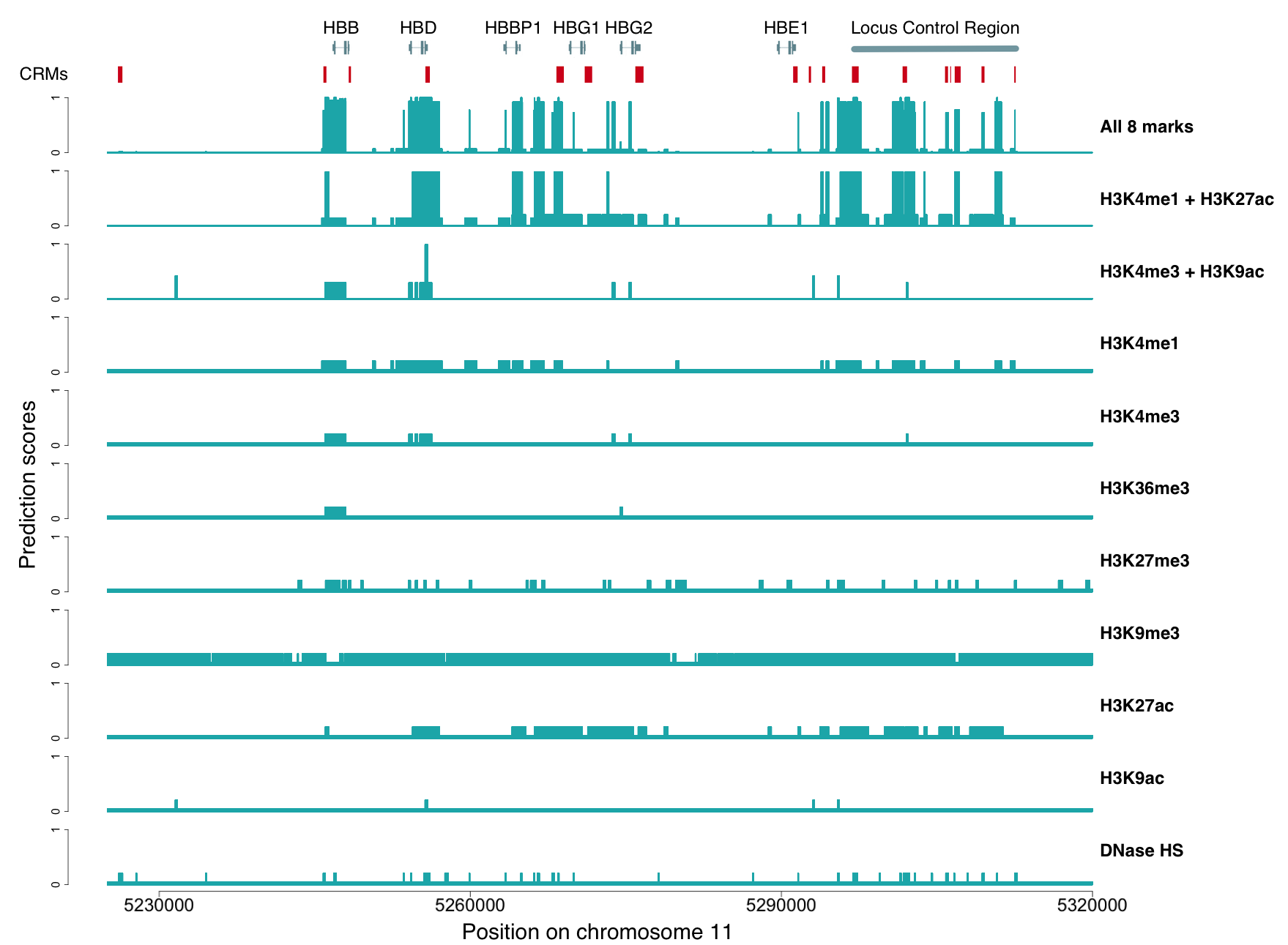

Supplement: S8 Fig — (TIFF) [file pgen.1005947.s009.tiff]

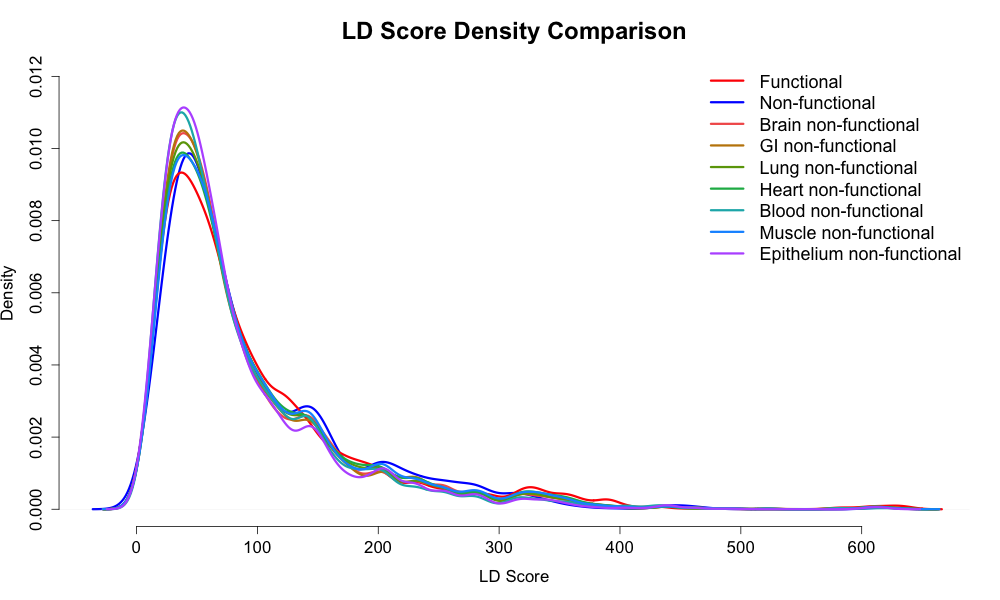

Supplement: S9 Fig — (TIFF) [file pgen.1005947.s010.tiff]
